# Identification of Genes Associated with Lemon Floral Transition and Flower Development during Floral Inductive Water Deficits: A Hypothetical Model

**DOI:** 10.3389/fpls.2017.01013

**Published:** 2017-06-13

**Authors:** Jin-Xue Li, Xiao-Jin Hou, Jiao Zhu, Jing-Jing Zhou, Hua-Bin Huang, Jian-Qiang Yue, Jun-Yan Gao, Yu-Xia Du, Cheng-Xiao Hu, Chun-Gen Hu, Jin-Zhi Zhang

**Affiliations:** ^1^College of Resources and Environment, Huazhong Agricultural UniversityWuhan, China; ^2^Institute of Tropical and Subtropical Cash Crops, Yunnan Academy of Agricultural SciencesKunming, China; ^3^Key Laboratory of Horticultural Plant Biology (Ministry of Education), College of Horticulture and Forestry Science, Huazhong Agricultural UniversityWuhan, China; ^4^College of Horticulture and Forestry Science, Huazhong Agricultural UniversityWuhan, China

**Keywords:** citrus, *FLOWERING LOCUS T*, flowering time, hormone, lemon, water deficit

## Abstract

Water deficit is a key factor to induce flowering in many woody plants, but reports on the molecular mechanisms of floral induction and flowering by water deficit are scarce. Here, we analyzed the morphology, cytology, and different hormone levels of lemon buds during floral inductive water deficits. Higher levels of ABA were observed, and the initiation of floral bud differentiation was examined by paraffin sections analysis. A total of 1638 differentially expressed genes (DEGs) were identified by RNA sequencing. DEGs were related to flowering, hormone biosynthesis, or metabolism. The expression of some DEGs was associated with floral induction by real-time PCR analysis. However, some DEGs may not have anything to do with flowering induction/flower development; they may be involved in general stress/drought response. Four genes from the phosphatidylethanolamine-binding protein family were further investigated. Ectopic expression of these genes in *Arabidopsis* changed the flowering time of transgenic plants. Furthermore, the 5′ flanking region of these genes was also isolated and sequence analysis revealed the presence of several putative *cis*-regulatory elements, including basic elements and hormone regulation elements. The spatial and temporal expression patterns of these promoters were investigated under water deficit treatment. Based on these findings, we propose a model for citrus flowering under water deficit conditions, which will enable us to further understand the molecular mechanism of water deficit-regulated flowering in citrus.

**Highlight:**

Based on gene activity during floral inductive water deficits identified by RNA sequencing and genes associated with lemon floral transition, a model for citrus flowering under water deficit conditions is proposed.

## Introduction

The transition from vegetative to reproductive growth is an important life-history event for flowering plants (Khan et al., 2014). Studies on the biology of flowering of model plants have identified several flowering regulatory pathways, such as the vernalization, photoperiod, circadian clock, age, autonomous, and gibberellin (GA) pathways (Khan et al., 2014). Different signaling pathways responding to endogenous and environmental signals converge on several floral integrator genes including *FLOWERING LOCUS T* (*FT*), *CONSTANS* (*CO*), *SUPPRESSOR OF OVEREXPRESSION OF CONSTANS 1* (*SOC1*), *FLOWERING LOCUS C* (*FLC*), *LEAFY* (*LFY*) (Wahl et al., 2013; Khan et al., 2014). Among them, *FT* encodes a mobile florigen signal. *SOC1* encodes a MADS box protein and regulates the expression of floral meristem identity genes (*LFY*), which links floral induction, inflorescence development and flowering (Liu et al., 2008). *FLC* is also a MADS box gene and strong suppressor of flowering (Michaels and Amasino, 1999). Other genes such as *SHORT VEGETATIVE PHASE* (*SVP*), *TERMINAL FLOWER 1* (*TFL1*), *TWIN SISTER OF FT* (*TSF*), and *BROTHER OF FT* and *TFL1* (*BFT*) are known to be suppressors of flower development and flowering (Guitton et al., 2012; Kazan and Lyons, 2016). Recent studies suggested that these genes are also key regulators of flower development and flowering time in citrus (Tan and Swain, 2007; Zhang et al., 2009a,b; Li et al., 2010; Chica and Albrigo, 2013a).

Traditionally, plant stress-regulated flowering is not formally recognized as a flowering pathway in past studies (Franks et al., 2007; Kazan and Lyons, 2016). However, several studies suggested that stress factors play key roles in controlling plant flowering (Chica and Albrigo, 2013a; Shanker et al., 2014; Kazan and Lyons, 2016). Water deficit is main stress factor that affects agricultural production, particularly irrigated land. For many annual and perennial plants, the emergence of flowering coincides with water deficit stress to ensure successful reproduction, a response known as “drought-escape” (Franks et al., 2007; Kazan and Lyons, 2016). Interestingly, flowering pathways play key roles in modulating drought tolerance (Riboni et al., 2013; Kazan and Lyons, 2016). Genetic knockouts in *Arabidopsis* have connected water deficit to ABA signaling as well as to several important genes in the flowering pathway (Kooyers, 2015). *GIGANTEA* (*GI*), *FT*, and *TSF* are key regulators of the drought-escape response (Riboni et al., 2013, 2016). Drought led to increased peak levels of *GI*. The mutant *gi* could not flower under drought stress (Riboni et al., 2013). The latest evidence suggests that ABA-dependent control of *GI* transcription enables “drought-escape” via up-regulation of *FT* expression in model plants (Riboni et al., 2016). *GI* does have other roles in carbon signaling and starch metabolism as well as interactions with the GA pathway (Putarjunan and Rodermel, 2014). Although genetic mechanisms connecting the drought response and flowering have been elucidated in model plants, how plants regulate flowering in response to drought remains poorly understood in woody plants.

Citrus is one of the most important and widely fruit crops (Tan and Swain, 2007; Xu et al., 2013). Citrus mainly bloom in the spring following the winter rest period in subtropical climates, similar to the normal response for temperate-zone deciduous fruit trees. However, the flowering of some important citrus varieties is induced during the dry season with flowering after the first effective rains of the rainy season in tropical regions (Chica and Albrigo, 2013a). Therefore, low-temperatures and water deficit are two key factors in flowering induction in sweet orange (Moss, 1969). Low-temperature (15°C) has been shown to directly affect the expression of *FT* homologs in Satsuma mandarin (Nishikawa et al., 2007). In sweet orange, *FT* responds rapidly (overnight) to floral inductive low-temperatures and requires alternation of light and dark cycles during induction (Chica and Albrigo, 2013b). In addition, some MADS box genes are also involved in the low-temperature regulation of flowering and bud dormancy in trifoliate orange typically *FLC* and *SVP* (Zhang et al., 2009a; Li et al., 2010). *PtFLC* was up-regulated expression during endodormancy (winter), and its expression is reduced after dormancy release (spring) in trifoliate orange (Zhang et al., 2009a). Similarly, *PtSVP* has also been involved in terminal bud formation and growth cessation (Li et al., 2010). Water deficit is another key floral inductive factor described in citrus (Southwick and Davenport, 1986; Chica and Albrigo, 2013a). For example, exposure to severe water deficit for 5 weeks produced maximum flowering intensity compared with milder deficit in Tahiti lime (*Citrus latifolia*) (Southwick and Davenport, 1986). A recent report suggested that water deficit induces flowering through the up-regulation of *FT* (Chica and Albrigo, 2013a). However, reports on the molecular mechanisms of floral induction and flowering by water deficit in citrus are still scarce. Therefore, enhanced understanding of the links between water deficit and flowering is essential for engineering drought tolerance in citrus.

To understand the impacts of water deficit on floral induction in lemon, we treated 1- to 2-year-old lemon [*C. limon* (L.) Burm. f.] trees cultivar (Femminello) and observed their morphological changes. We also examined gene expression by RNA sequencing, identifying 1000s of genes potentially involved in water deficit responses in flowering induction. In particular, we found that four genes from the phosphatidylethanolamine-binding protein (PEBP) family were important for flowering of lemon. Together, the results provide evidence for regulation of citrus floral induction under water deficit conditions and establish a foundation for advanced research on functional flowering genes in citrus and similar plants.

## Materials and Methods

### Plant Material and Experimental Conditions

All plants were grown in the greenhouse of the Institute of Tropical and Subtropical Cash Crops, Yunnan Academy of Agriculture Sciences. We used 2-year-old lemon trees (Femminello) propagated by bud grafting to trifoliate orange rootstocks, 80 and 15 healthy trees were selected for water deficit-treated and control, respectively. The trees ranged in height from 1 to 1.5 m growing in 52-cm plastic pots containing potting mix of commercial medium and perlite (3:1). Trees in the greenhouse were exposed to natural variations in photoperiod throughout the experiment during Summer (from June to July) 2015. Before the water deficit treatment, trees were kept in a greenhouse and watered every 2 days to saturation. Minimum and maximum temperatures were 23 and 31°C, respectively. At the beginning of the water deficit, trees were fully watered and soil moisture was measured 4 h later by Theta probe type ML2x (Delta-T Devices Ltd., Cambridge, England), and the soil water naturally evaporated until reaching the set level (around 15%). Then the soil moisture was maintained by accurately watering according to the daily loss of water (pots were weighed and watered thrice per day). Leaf water potential was measured with a pressure chamber (PMS Instrument Co., Corvallis, OR, United States), thrice per day (10:00 am, 2:00 pm, and 6:00 pm). After 2 weeks, trees were fully watered. In this study, the soil moisture was measured three times per pot and leaf water potential was detected once per plant with the values indicated as the mean ± SE of three plant leaves, all treated plants were investigated. When the plants were fully watered, the soil moisture was about 30%, and the corresponding leaf water potential was near –1 MPa. Under water deficit treatment, soil moisture levels were maintained at around 15% corresponding to approximately 50% of the control, and the corresponding water potentials were around -1.5 MPa. In this study, terminal bud and the following five buds from flushes were collected at three stages (stage 1: 1 week before water deficit; stage 2: 1 week after the beginning of water deficit; and stage 3: 1 week after releasing from water deficit). It is worth noting that flower buds were visible to the naked eye at stage 3. To analyze the expression of *CiFT*, leaf sample was also collected. Only mature healthy fully expanded leaves were randomly collected from the three most apical nodes of shoots. Considering that *CiFT* might be affected by light, all leaves were sampled at 10:00 am. In this study, bud and leaf samples were collected from three groups of trees used as three biological repeats, each group containing three trees. All plant tissues were sampled, immediately frozen in liquid nitrogen, and stored at –80°C until used.

**FIGURE 1:**
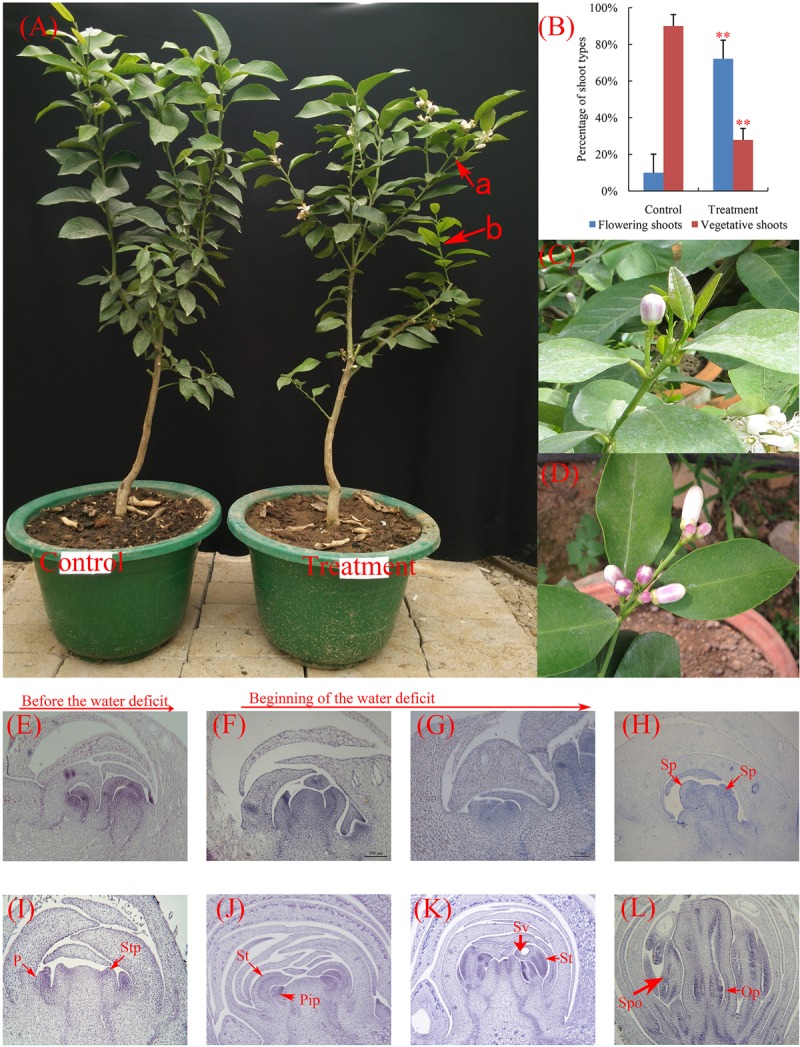
Phenotypic characteristics and cytological changes of lemon during floral inductive water deficits. **(A)** The control and water deficit-treated plants after resumption of irrigation. a, a statistical unit of flowering shoot; b, a statistical unit of vegetative shoot. **(B)** Effect of water deficit on flowering of lemon. The data were processed using one-way analysis of variance (ANOVA), and statistical differences were compared based on Student’s *t*-test, taking *P* < 0.01 as ^∗∗^. **(C)** Single flowers from the water deficit-treated plants. **(D)** Leafless inflorescences from the water deficit-treated plants. **(E–L)** The floral differentiation on lemon buds. The integrated flower bud was formed in 2 or 3 weeks, and then flower bud began flowering. **(E)** Undetermined state. **(F)** Lateral bud growth resumes. **(G,H)** Flower primordium forms and sepal primordia arise. Sp, sepal primordia; P, petal; **(I,J)** Stamen primordia arise. Stp, stamen primordia; Pip, pistil primordia; **(K)** Stamen and pistil primordia arise. St, stamen; Sv, secretion vesicle. **(L)** Fully developed floral bud. Spo, sporogenous; Op, ovule primordia. The bars represent 50 μm **(E–H)** and 100 μm **(I–L)**.

For morphological observation, 15 water deficit-treated and 15 untreated trees were selected and tagged, respectively. For flowering evaluation, each shoot as a statistical unit (**Figure 1A**). All shoots of each tree were counted, no flowering shoots as vegetative shoots. The data were processed using one-way analysis of variance (ANOVA), and significant differences were compared based on Student’s *t*-test. *P* < 0.01 was considered significant. Paraffin section analysis was performed as described by Yao et al. (2007), approximately 500 buds were selected and tagged under a similar growing condition. Ten shoots from lemon derived from these buds were collected every 4 days before the water deficit treatment and every 2 days beginning of water deficit.

### Hormone Treatments on Lemon Trees

Trees were randomly divided into five groups with 15 plants per group and subjected to treatments in a growth chamber with adjustable temperature (Minimum and maximum temperatures were 23 and 31°C, respectively). These trees were treated by spraying 40 mg L^-1^ of GA_3_, 100 mg L^-1^ of ABA, 40 mg L^-1^ of NAA, and 800 mg L^-1^ of paclobutrazol (PBZ, a GA biosynthesis inhibitor) onto entire trees with a hand-gun sprayer, using approximately 0.5 L per tree and wetting the tree to the point of run-off. Meanwhile, a non-ionic wetting agent (Tween 20, 20% w/v) was added at a rate of 0.05% v/v in all treatments. Water-treated trees served as control. Samples were collected at 15-days intervals until flower bud formation of PBZ-treated trees (visible to the naked eye). The *CiFT* expression was measured in leaves, whereas other genes were measured in buds.

### RNA Extraction and Sequencing

Total RNA was extracted as previously described (Zhang et al., 2009b). The libraries were produced and sequenced using Illumina’s Genome Analyzer (Solexa; Illumina, San Diego, CA, United States). The sequencing and data analysis were carried out essentially as described in previous studies (Mortazavi et al., 2008; Conesa et al., 2016). In this study, three biological replicates were carried out for the RNA Seq analysis. Raw sequence reads were filtered for quality using the FASTX-Toolkit (Blankenberg et al., 2010). All clean reads were mapped to the citrus genome^1^ (Xu et al., 2013) using Burrows–Wheeler Aligner (BWA) software (Li and Durbin, 2010). In this study, the NOIseq was used to identify differentially expressed genes, probability ≥ 0.8 and the absolute value of log_2_^Ratio^ ≥ 1 were used as the threshold (Tarazona et al., 2011). Gene annotation was conducted using the Blast2GO program (Conesa et al., 2005). The alternative splicing (AS) events from different stages were identified using TopHat (Trapnell et al., 2009). All the junction sites of the same gene were used to distinguish the type of AS events, including alternative 5′-splice sites (A5SS), alternative 3′-splice sites (A3SS), skipped exons (ES), and retained introns (RI). During the analysis of the AS *CiFT*, we found that two PCR products were generated by a pair of *CiFT* primers, implying the existence of *CiFT* AS in lemon. By comparing the two *CiFT* cDNA sequences, we discovered that one splicing product contained 88 amino acids of open reading frame (ORF) because of intron 2 and 3 retention compared with a typical *CiFT* gene. Thus, we designated this form as *CiFT2*β and the full-size transcript as *CiFT2*α.

### Real-Time PCR Verification

Real-time PCR was performed with SYBR Green I chemistry (Qiagen, Hilden, Germany) as described previously (Zhang et al., 2009b, 2014). All primers are listed in **Supplementary Table S1**. Data were evaluated by using the LightCycler 480 software version 1.5 (Roche Applied Science, Mannheim, Germany) and normalized to expression of β-*actin* (Zhang et al., 2014). Three independent biological replications of each sample and four technical replications of each biological replication were used for real-time PCR.

### Quantification of Hormones

The samples for ABA, IAA, and GA_3_ quantification were prepared according to a previously reported protocol (Pan et al., 2010; Wu et al., 2014). The Icon Isotopes of internal standard (d_6_-ABA, d_5_-IAA, and d_2_-GA_3_) were used for ABA, IAA, and GA_3_, respectively. An Agilent 1100 HPLC (Agilent Technologies, Santa Clara, CA, United States), C18 column (150 mm × 2.1 mm, 5 μm; Waters, Milford, MA, United States), and API3000 MS-MRM (Applied Biosystems, Foster City, CA, United States) were used for the analysis (Pan et al., 2010; Wu et al., 2014). Three biological replications were assayed for each sample in this study.

### *Arabidopsis* Transformation and Histochemical Localization of *GUS* Activity

To generate the over-expression vectors of *CiFT*α, *CiFT*β, *CiBFT*, *CiTFL1*, and *CiMFT*, each full-length cDNA sequence was cloned into the binary vector pBI121 with the ClonExpress One Step Cloning Kit (Vazyme Biotech Co., Ltd., Nanjing, China) according to the manufacturer’s instructions, respectively. Meanwhile, the promoter of *CiFT*, *CiBFT*, *CiTFL1*, and *CiMFT* was also cloned into the DX218 vector, respectively. The floral dipping transformation method was used in this experiment (Clough and Bent, 1998). The surviving plants were transplanted into soil and grown under long-day conditions (16 h light/8 h dark) at 22 ± 1°C. Morphological analyses were investigated in the transgenic plants T_3_. The number of rosette leaves and days to flowering were counted when transgenic plants bore a 1-cm-long inflorescence (Li et al., 2010; Sun et al., 2014).

### Water Deficit Treatment and Histochemical Localization of GUS Activity in *Arabidopsis*

Seeds from transgenic plants T_3_ were vernalized in the dark at 4°C for 1 week before sowing. Approximately 200 vernalized seeds were sown on a plastic plate (9-cm), then the plates with seeds were transferred into a Percival growth chamber (Percival Scientific, Percival AR41L2), which was set at standard conditions (22°C, 60% humidity, 16 h light/8 h dark, ∼300 μmol m^-2^ s^-1^ photon flux) until two-leaf stage. When the seedlings reach two-leaf stage, extra seedlings were removed, leaving approximately 100 plants per plate under similar growth status. For the drought treatments, the relative air humidity in the chamber was adjusted to 30% to accelerate the water evaporation at the beginning of the water deficit. Meanwhile, treated plants stop watering and soil moisture was measured by Theta probe type ML2x. Soil moisture levels were maintained at around 15% corresponding to approximately 50% of the control. After 3 days, whole plant material was collected. GUS staining was performed as previously described (Zhang et al., 2014). In this study, three independent transgenic lines for each promoter were analyzed.

## Results

### Flowering Response Induced by Water Deficit Treatment

Lemon trees produced more flowers than control trees under water deficit condition (**Figure 1A**). Under control condition, very few flowering shoots (10% of total shoots) were produced in irrigated trees, whereas 72% of shoots flowered when trees were exposed to water deficit (**Figure 1B**). Most of the flowers formed in trees were of the axillary flower under water deficit condition (flower from axillary buds, **Figures 1C,D**). These results indicated that the water deficit treatment was effective method at inducing lemon flowering.

To examine cytological changes of buds during floral inductive water deficits, buds were collected, fixed, and stained with hematoxylin for microscopic examination (**Figures 1E–L**). The paraffin sections analysis of buds showed that lemon do not flower bud differentiation, begin to produce vegetative buds before water deficit (**Figure 1E**). Under water deficit conditions, the floral buds rapidly initiated differentiation (about 3–4 days after the beginning of water deficit, **Figures 1F–H**). Floral development hastened differentiation and produced the primordia of the floral organ including sepal, petal, stamen, and pistil (**Figures 1H–L**). The whole integrated flower bud was formed in 2 or 3 weeks, and then part of the flower bud began to flowering.

### Analysis of Dynamic Changes in Bud Transcriptome with RNA Sequencing

To analyze dynamic changes in the lemon bud transcriptome during floral inductive water deficits, RNA sequencing was performed on lemon buds at three stages (stage 1: 1 week before water deficit; stage 2: 1 week after the beginning of water deficit; and stage 3: 1 week after releasing from water deficit). After removing low-quality reads, approximately 28 million clean reads were obtained for each biological replicate. From stage 1 to stage 2, of the 21,947 read-mapped genes detected, more genes were up-regulated (11,490) than down-regulated (10,457). From stage 2 to stage 3, of the 21,763 read-mapped genes detected, more genes were down-regulated (11,956) than up-regulated (9807). Similarly, of the 21,947 read-mapped genes detected from stage 1 to stage 3, more genes were down-regulated (11,055) than up-regulated (10,892). A total of 22,354 non-redundant genes were expressed during floral inductive water deficits when the data from the three stages were combined. Of these, 406 were not observed from stage 1 to stage 2, 326 were not found from stage 2 to stage 3, and 591 were not found from stage 1 to stage 3.

.

### Large-Scale Identification of Alternative Splicing by RNA Sequencing

**FIGURE 2:**
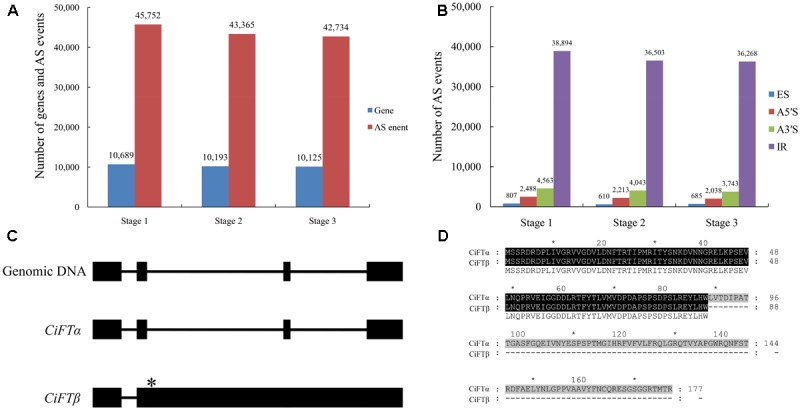
Large-scale identification of alternative splicing (AS) by RNA sequencing during floral inductive water deficits. **(A)** The AS events and genes at different stages (stage 1: 1 week before water deficit; stage 2: 1 week after the beginning of water deficit; and stage 3: 1 week after releasing from water deficit). **(B)** The different types of the AS events at different stages (stage 1: 1 week before water deficit; stage 2: 1 week after the beginning of water deficit; and stage 3: 1 week after releasing from water deficit). ES, skipped exons; RI, retained introns; A5SS, alternative 5′-splice sites; A3SS, alternative 3′-splice sites. **(C)** The structural comparison between the *CiFT* genomic sequence and putative AS transcripts of *CiFT* (*CiFT*α and *CiFT*β), asterisk indicates location of termination codon. **(D)** The sequence analysis of CiFTα and CiFTβ protein.

To explore potential AS events during floral inductive water deficits, gene structure analyses of AS genes were performed. When combining cDNA of the sweet orange genome (Xu et al., 2013) and RNA sequencing data, more than 27% (12,041) of the multi-exon genes contained at least one AS event. In this study, we detected 45,752 splice junctions in 10,689 genes at stage 1, 43,365 splice junctions in 10,193 genes at stage 2, and 42,734 splice junctions in 10,125 genes at stage 3 (**Figure 2A**). We also identified the following four common types of AS: A3SS, A5SS, ES, and RI. At stage 1, classification of the 45,752 splice junctions showed that 807 (1.8%) splice junctions corresponded to ES, 2488 (5.4%) corresponded to A5S, 4563 (10.0%) corresponded to A3S, and 38,894 (85.0%) corresponded to IR. At stage 2, of the 43,365 splice junctions 610 (1.4%) splice junctions corresponded to ES, 2213 (5.1%) corresponded to A5S, 4043 (9.3%) corresponded to A3S, and 36,503 (84.2%) corresponded to IR. At stage 3, of the 42,734 splice junctions 685 (1.6%) splice junctions corresponded to ES, 2038 (4.8%) corresponded to A5S, 3743 (8.6%) corresponded to A3S, and 36,268 (84.9%) corresponded to IR (**Figure 2B**). Intron retention was the most prevalent mechanism. There might be two possible explanations for the high percentage of IR transcripts: at first, some novel transcriptional events may have occurred during under water deficit condition. Secondly, the ORF from reference transcripts may be inaccurate because their ORFs were predicted based on triplet code (Xu et al., 2013).

We noted many AS events of flowering-related genes, such as *RELATIVE OF EARLY FLOWERING 6* (*REF6*), *FLC*, *FCA*, *EMBRYONIC FLOWER 1* (*EMF1*), *FT*, and *FY*. Almost all reported AS events of citrus flowering time genes were found, including *FLC*, *FCA*, and *FY* (Zhang et al., 2009a; Ai et al., 2012, 2016). Here, we report the splicing of lemon *FT* (*CiFT*) as an example. *CiFT* comprises four exons and three introns according to DNA analysis of lemon; two AS forms were detected based on RNA sequencing. Further expression analysis of *CiFT* was conducted by reverse transcriptase PCR (**Figure 2C**). Two AS transcripts of *CiFT* were isolated from lemon. One of the AS transcripts showed high identity with trifoliate orange and *Citrus unshiu FT* and was named *CiFT*α, and only some SNPs (single nucleotide polymorphism) were found compared with the protein-coding sequences of the previously published trifoliate orange and sweet orange *FT* (Kobayashi et al., 1999; Endo et al., 2005; Zhang et al., 2009b). The other AS transcript contains four exons and two introns (because of intron 2 and 3 retention) and was named *CiFT*β. The isolated cDNA of *CiFT*β is 2429 bp long, and 267 nucleotides of an ORF contained 88 amino acids based on bioinformatics prediction (**Figure 2D**) because the second intron has a termination codon (**Figure 2C**).

### Differential Transcriptome Responses of Bud under Water Deficit Conditions

**FIGURE 3:**
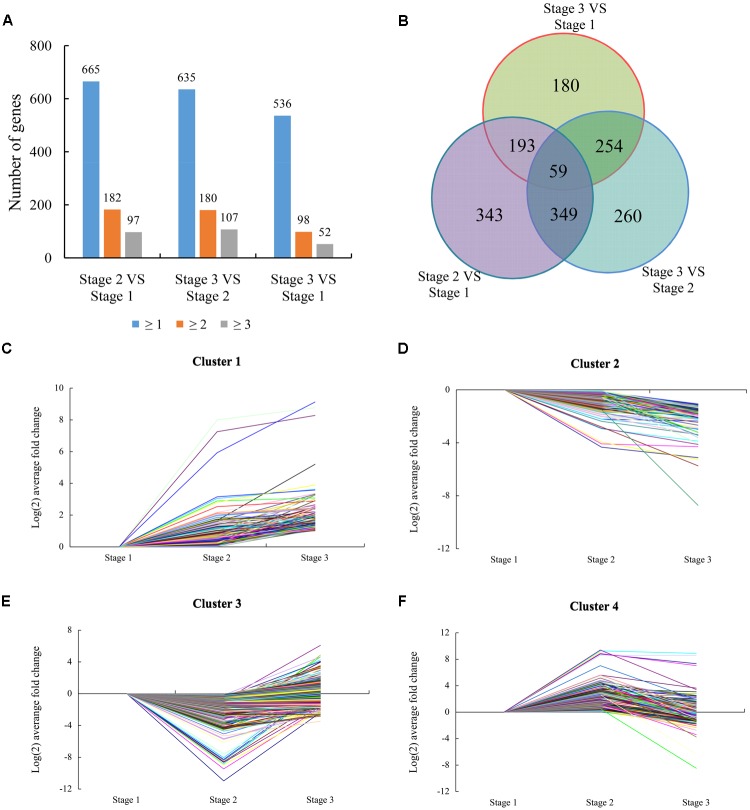
Expression patterns of differentially expressed genes (DEGs). **(A)** The total numbers of genes differentially expressed (including up- or down-regulated, fold changes ≥ 1, 2, and 3) at stage 2 compared with stage 1, stage 3 compared with stage 2, and stage 3 compared with stage 1, respectively. **(B)** Venn diagram showing the overlapping of DEGs at three different stages (Stage 2 compared with stage 1, stage 3 compared with stage 2, and stage 3 compared with stage 1). **(C)** Cluster 1 consisting of 172 DEGs. **(D)** Cluster 2 consisting of 181 DEGs. **(E)** Cluster 3 consisting of 776 DEGs. **(F)** Cluster 4 consisting of 509 DEGs.

To identify flowering-related genes, the number of normalized gene reads of different stages was calculated using FPKM. Genes were considered to be differentially expressed based on probability ≥0.8 and an absolute value of log_2_^Ratio^≥1 as a threshold (Tarazona et al., 2011). According to these criteria, 944 genes were DEGs from stage 1 to stage 2, and 455 were up-regulated and 489 were down-regulated. From stage 2 to stage 3, 922 genes were DEGs, and 596 were up-regulated and 326 were down-regulated. From stage 1 to stage 3, 686 DEGs were identified, with 395 up-regulated and 291 down-regulated (**Figure 3A**). By combining results from the three stages, 1638 DEGs were identified as candidates that may represent the common flowering-related genes, and 59 DEGs were shared in all three stages that may represent the common flowering-responsive genes (**Figure 3B**). And then, a homology search was conducted using the NCBI database to investigate the biological processes possibly regulated by the 1638 DEGs (**Supplementary Table S2**). We detected 1519 DEGs as having homology with known proteins and the remaining 19 did not in the NCBI database. Based on Gene Ontology annotation of these genes performed by Blast2GO analysis, 1436 DEGs were divided into the three principal GO organization categories (**Supplementary Figure S1**): molecular function, biological process, and cellular components.

Based on the similarity of the expression patterns of the DEGs, 1638 DEGs were classified into four clusters. The gene from cluster 1 (including 172 genes) was induced at stage 1 and most maintained high expression levels at stage 3 compared with stage 1 and stage 2 (**Figure 3C**). This cluster featured genes encoding transcription factors (TFs), biotic/abiotic response proteins, and heat shock proteins. These genes were significantly induced at the beginning of water deficit, indicating that the gene cluster might play a key role in the necessary development of lemon. The gene from cluster 2 (including 181 genes) was suppressed at stage 1 and maintained low expression levels at stage 3 compared with stage 1 and stage 2 (**Figure 3D**). These genes were involved in transcriptional regulation, protein metabolism, and ABA signaling, according to BLAST analysis. These genes may involved in meristem gene regulation and development of vegetative buds. Meanwhile, hormone-related genes and stress proteins were featured in this cluster, indicating these genes might be related to the water deficit response. The gene from cluster 3 (including 776 genes) was transiently suppressed at stage 2 and was then induced at stage 3 (**Figure 3E**). This cluster also featured genes encoding hormone (GAs, ABA, auxin, and ethylene) signaling/biogenesis and flowering control proteins. These genes shows up-regulated at stage 3, indicating these genes involved in flowering and recovery of vegetative growth. The gene from cluster 4 (including 509 genes) that was transiently induced at stage 2 and was then suppressed at stage 3 (**Figure 3F**). The suppression of the gene cluster may imply possible involvement in drought stress response, floral induction, and flower bud differentiation of lemon.

### Identification of Flowering-Related Genes by RNA Sequencing

Many previously reported flowering-related genes were found among DEGs such as *GI*, *FLC EARLY FLOWERING 3* (*ELF3*), and *EARLY FLOWERING 4* (*ELF4*) (**Supplementary Table S2**). The expression pattern of these genes was closely correlated with flowering during floral inductive water deficits. In *Arabidopsis*, drought and ABA promote transcriptional up-regulation of PEBP family members such as *FT* and *TSF* leading to flowering under long days (Riboni et al., 2013). In this study, the two genes showed high identity with *MOTHER OF FT* (*MFT*) and *BFT* from the PEBP gene family. Moreover, some additional related flowering genes that have not been placed in any specific flowering pathway were also identified in this study, such as the *SQUAMOSA PROMOTER BINDING PROTEIN* family genes (*SPL6/8/9/13*) and MADS TF family genes. Many genes involved in different hormone synthesis and signaling pathways showed significant expression changes during floral inductive water deficits (**Supplementary Table S2**). For example, four ABA-related genes (two ABA 8-hydroxylase genes and two ABA stress-related proteins) showed significant up-regulation under water deficit condition, which encode key enzymes in ABA biosynthesis and metabolism. Eleven auxin-related genes were significant altered included four auxin-induced proteins, three auxin response factors, three auxin transporters, and one auxin canalization protein. The auxin response factors were down-regulated and auxin-induced proteins were up-regulated at the beginning of water deficit. After the water deficit treatment, cluster 3 genes for ethylene biosynthesis and perception were up-regulated, including 15 ethylene-responsive TFs and 1 ethylene response protein. In addition, six genes involved in the response to GA stimulus pathways were up-regulated at the beginning of water deficit. Furthermore, the biological interpretation of the DEGs was further investigated by KEGG pathway analysis. The most frequently represented pathways are plant hormone signal transduction and metabolic pathways at different stages (**Supplementary Figure S2**). These results suggest that plant hormone are involved in the regulation of flowering under water deficit conditions.

### Verification of the DEGs

**FIGURE 4:**
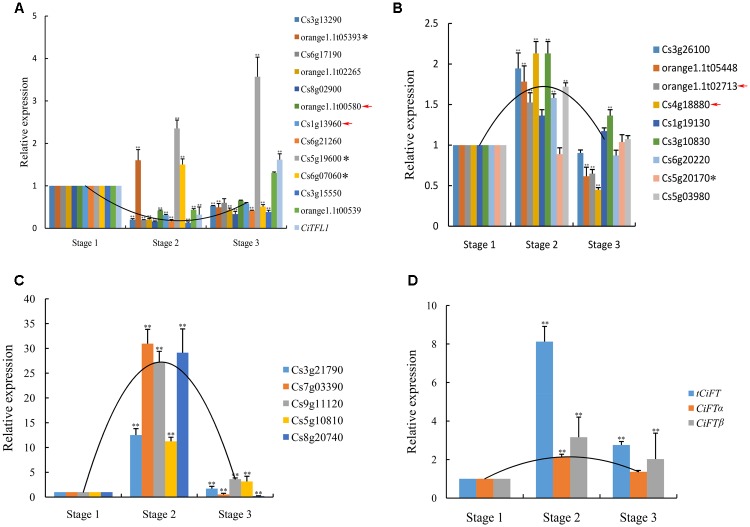
Real-time PCR confirmation of the DEGs. **(A)** The expression pattern of 14 genes belong to cluster 3 were verified. **(B)** Nine low levels of genes belonging to cluster 4 were verified. **(C)** Five high levels of genes belonging to cluster 4 were verified. **(D)** Transcript level of *CiFT* during floral inductive water deficits. Asterisk indicates that these genes showed different expression patterns between real-time PCR and RNA sequencing. Red arrow indicates that these genes of no significantly differential expression at different stages by RNA sequencing. Relative transcript levels are calculated by real-time PCR with *Actin* as a standard. Three independent biological replications of each sample and three technical replications of each biological replication were used for real-time PCR analysis, and all biological replications showed similar trends. Data from one biological replication are presented, data were indicated as means ± SD (*n* = 4). The data were processed using ANOVA, and statistical differences were compared based on Student’s *t*-test, taking *P* < 0.01 as ^∗∗^.

To validate the expression profiles obtained by RNA sequencing, a total of 26 genes (The expression pattern of 14 genes belong to cluster 3 and 12 genes belong to cluster 4) were chosen to real-time PCR analysis; these genes included 24 DEGs, four genes of no differential expression. The results from real-time PCR were compared with RNA sequencing data. For 22 of the 26 genes, the same expression patterns was shown between the RNA sequencing data and real-time PCR analysis (**Figures 4A–C**), the remaining four genes showed different expression patterns according to RNA sequencing and real-time PCR.

In this study, the expression of *CiTFL1* was not detected during floral inductive water deficits. One possible explanation is that its mRNA levels were too low to be detected by RNA sequencing. These results consistent with our previous reports on *TFL1* in citrus (Zhang J.Z. et al., 2011). Therefore, the expression of lemon *CiTFL1* was investigated in lemon bud by real-time PCR. The expression of *CiTFL1* was transiently suppressed under water deficit treatment and was then induced at the resumption of irrigation. On the other hand, citrus *FT* was mainly expressed in leaves, with little or no expression in citrus buds based on our previous study (Zhang et al., 2009b). Therefore, the expression of lemon *FT* (*CiFT*α; *CiFT*β; *tCiFT*: Total *CiFT* including *CiFT*α and *CiFT*β) was investigated in lemon leaves, and the results showed that the accumulation of *tCiFT* significantly increased under water deficit treatment (**Figure 4D**). However, when water deficit was interrupted, the expression levels returned to the initial levels. The expression patterns of *CiFT*α and *CiFT*β were similar to that of *tCiFT*. These results indicated that the expression of *CiFT* was induced by water deficit.

### Quantification of Endogenous Plant Hormones and Different Hormone Treatments

**FIGURE 5:**
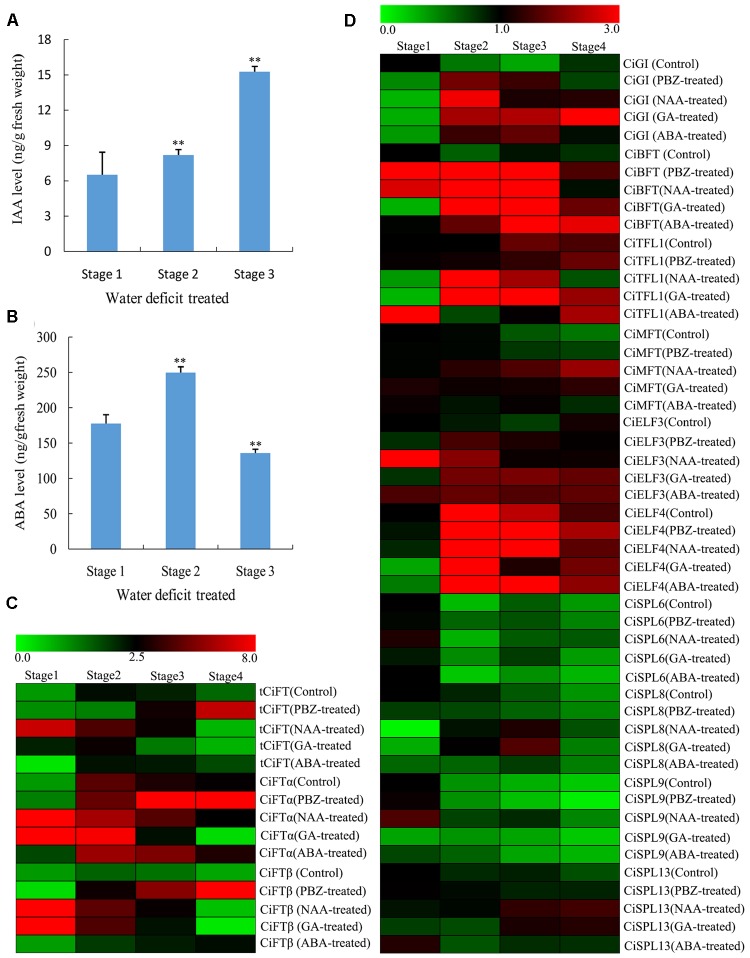
Changes of different hormone contents of lemon buds during floral inductive water deficits and the expression pattern of DEGs by different hormone treatments. **(A)** Changes in IAA content (ng/g fresh weight) in lemon buds (Stage 1: 1 week before water deficit; stage 2: 1 week after the beginning of water deficit; and stage 3: 1 week after releasing from water deficit). The data were processed using ANOVA, and statistical differences were compared based on Student’s *t*-test, taking *P* < 0.01 as ^∗∗^. **(B)** Changes in ABA content (ng/g fresh weight) in lemon buds (Stage 1: 1 week before water deficit; stage 2: 1 week after the beginning of water deficit; and stage 3: 1 week after releasing from water deficit). The data were processed using ANOVA, and statistical differences were compared based on Student’s *t*-test, taking *P* < 0.01 as ^∗∗^. **(C)** Heat map showing the expression of total CiFT (*tCiFT*), *CiFT*α, and *CiFT*β in plant lemon leaves treated with PBZ, NAA, GA_3_, and ABA treatments. Plant materials from PBZ-treated, GA_3_-treated, ABA-treated, NAA-treated, and water-treated trees were collected at four stages (stage 1, stage 2, stage 3, and stage 4). **(D)** Heat map showing the expression of *CiGI* (Cs3g21790), *CiBFT* (Cs8g15080), *CiMFT* (Cs2g06960), *CiELF3* (Cs1g19130), *CiELF4* (Cs7g31110), *CiSPL6* (Cs5g12260), *CiSPL8* (Cs1g03630), *CiSPL9* (orange1.1t02265), and *CiSPL13* (orange1.1t02597) in lemon buds during PBZ, NAA, GA_3_, and ABA treatments. Plant materials from PBZ-treated, GA_3_-treated, ABA-treated, NAA-treated, and water-treated trees were collected at four stages (stage 1, stage 2, stage 3, and stage 4). Three independent biological replications of each sample and four technical replications of each biological replication were used for real-time PCR analysis. Genes highly or weakly expressed in the tissues are colored red and green, respectively. The heat map was generated using Cluster 3.0 software.

The RNA sequencing data revealed that hormone-related genes changed greatly during the water deficit process, especially genes for ABA biosynthesis and metabolism, followed by the genes for GA and IAA biosynthesis and signal transduction. Therefore, the ABA, IAA, and GA_3_ contents of buds were measured in water deficit-treated and control trees. IAA increased immediately at the beginning of the water deficit treatment and maintained high expression levels until the resumption of irrigation (**Figure 5A**). ABA content was transiently induced during floral inductive water deficits and was then suppressed at resumption of irrigation (**Figure 5B**). Unfortunately, we were unable to detect GA_3_ successfully. Therefore, trees were sprayed with PBZ, a GA biosynthesis inhibitor, and untreated trees served as the control. The number of flowers in the PBZ-treated trees was increased compared to the control (**Supplementary Figure S3**). PBZ yielded a similar trend with water deficit treatment. These results indicate that the GAs content may be suppressed during water deficit.

To investigate the effect of exogenous hormones on citrus flowering and the expression of flowering genes, GA_3_, ABA, and NAA were also sprayed onto entire trees. Some new vegetative shoots were formed by GA_3_ and NAA treatment compared with the control. However, no significant differences were observed by ABA treatment. Samples were collected at 15-days intervals until flower buds of PBZ-treated trees were visible to the naked eye. In this study, plant materials from PBZ-treated, GA_3_-treated, ABA-treated, NAA-treated, and water-treated trees were collected at 4 stages (stages 1, stage 2, stage 3, and stage 4). It appears that stage 1 was the bud induction period and stage 2 and 3 were flower bud differentiation period because part of the flower bud from PBZ-treated trees were visible to the naked eye at stage 4 compared with the control trees. The expression of related flowering DEGs (*tCiFT*, *CiBFT*, *CiTFL1*, *CiELF*, and *CiSPLs*) was investigated at different treated stages. Compared with the control trees, the expression of *tCiFT* in leaves was significantly increased by ABA and PBZ treatment (**Figure 5C**). The expression level of *tCiFT* in GA_3_- and NAA-treated trees was significantly suppressed from stage 3 to stage 4. Under PBZ treatment condition, the expression patterns of *CiFT*α and *CiFT*β were similar to that of *tCiFT*, indicating that they may perform a similar function role during the treatment process. For different hormone treatments, the relative expression of *CiGI*, *CiTFL1*, and *CiBFT* showed significantly higher values throughout the entire treated period (**Figure 5D**). They increased at the beginning of the treatment, then maintaining a high level of expression during treatment, and tended to decrease at the end of the treatment. The levels of *CiBFT1* and *CiTFL1* accumulation in buds were induced during the entire treatment period by ABA. A high transcript level of *CiMFT* was seen as the beginning of treatment, and it was then maintained at a low level except when treated with NAA. No differences in the expression of *CiELF3* and *CiELF4* were detected between the control and the treated trees; they showed high expression levels throughout the entire period studied. The relative expression levels of *CiSPL6, CiSPL8*, and *CiSPL9* were not significantly altered. They were detected and present at low levels during the treatment process, and their expression levels were independent of treatments throughout the study period. Relative expression of *CiSPL13* was different from other *CiSPL* genes at stage 4; the accumulation of *CiSPL13* was induced at stage 4 by NAA and GA treatments (**Figure 5D**).

### Functional Analysis of *CiFT*α, *CiFT*β, *CiBFT*, *CiTFL1*, and *CiMFT* in Transgenic *Arabidopsis*

**FIGURE 6:**
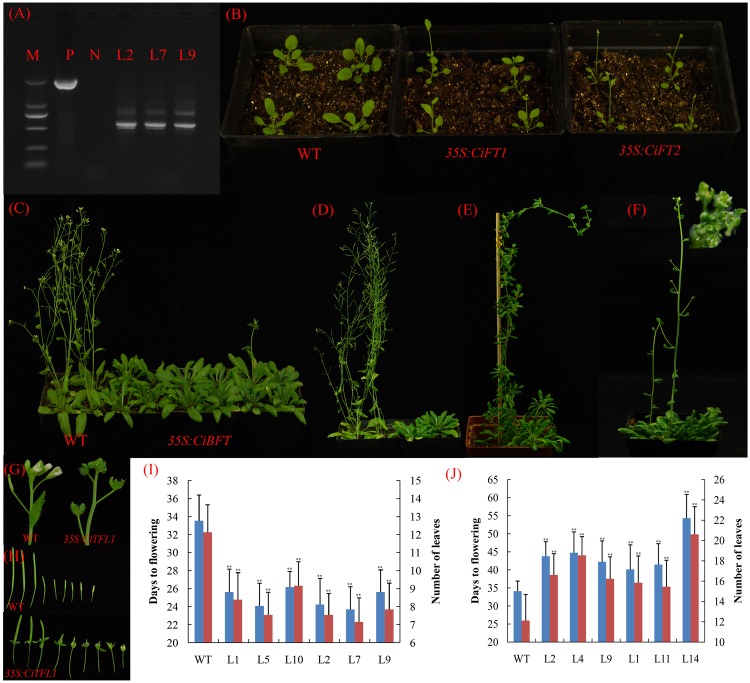
Phenotypes of *35S::CiFT*α, *35S::CiFT*β, *35S::CiBFT*, and *35S::CiTFL1* transgenic *Arabidopsis*. **(A)** RT-PCR analysis of the accumulation of *CiFT*β in *35S::CiFT*β transgenic *Arabidopsis* RNA level. P: positive plasmid as a control, N: wild type *Arabidopsis* as a negative control, M: Marker 5000; L2, L7, and L9 are the *35S::CiFT*β transgenic lines. **(B)** Two randomly selected lines that constitutively expressed *35S::CiFT*α and *35S::CiFT*β exhibited an early-flowering phenotype relative to wild-type controls under long days. **(C)** Ectopic expression of *CiBFT* in wild-type *Arabidopsis* delayed the flowering under long days. **(D)** Ectopic expression of *CiTFL1* in wild-type *Arabidopsis* delayed flowering. **(E)** Two transgenic lines from *35S::CiTFL1* plants flowered significantly later than the wild-type plants. **(F)** Floral defects phenotype with conversion of sepals into leaf-like structures in some *35S:: CiTFL1* lines. **(G)** Severe phenotype with conversion of sepals into leaf-like structures in some *35S:: CiTFL1* lines. **(H)** Persistent sepals around the fruits in some *35S:: CiTFL1* lines. **(I)** Number of leaves and times to flowering of T_3_ plants of six independent transgenic lines from *35S::CiFT*α (L1, L5, and L10) and *35S::CiFT*β (L2, L7, and L9). Blue bar indicates number of days to flowering, red bar indicated number of leaves to flowering. **(J)** Number of leaves and times to flowering of T_3_ plants of six independent transgenic lines from *35S::CiBFT* (L2, L4, and L9) and *35S::CiTFL1* (L1, L11, and L14). Blue bar indicates number of days to flowering, red bar indicated number of leaves to flowering. The data were processed using ANOVA, and statistical differences were compared based on Student’s *t*-test, taking *P* < 0.01 as ^∗∗^.

To assess the functional characteristics of *CiFT*α, *CiFT*β, *CiBFT*, *CiTFL1*, and *CiMFT*, these genes were over-expressed in *Arabidopsis*. Sixteen, 20, 18, 14, and 26 independent T_1_ transgenic lines were generated, respectively. Interestingly, *CiFT*β transcribed several new transcripts (**Figure 6A**). Comparison of the various *CiFT*β cDNA revealed that four AS transcripts of *CiFT*β were identified; Among these AS transcripts, one of the transcripts was the same as *CiFT*α, other transcripts could not encode complete protein compared with *CiFT*α because of intron retention or A3SS (**Supplementary Figure S4**).

To further analyze the function of these genes, three transgenic lines were randomly selected for each gene. We selected 15 T_3_ plants for each transgenic line. Compared with control plants, the *35S::CiFT*α and *35S::CiFT*β transgenic lines flowered significantly earlier than control plants in terms of both number of leaves and days to flowering (**Figure 6B**). In *35S::CiFT*α and *35S::CiFT*β, the average time to flowering ranged from 23.7 to 26.1 days in six transgenic lines, whereas that of the control plants was 33.5 days. The average number of leaves at flowering ranged from 7.1 to 9.1 and was 12.1 control plants (**Figure 6I**). The *35S::CiFT*β flowered earlier than *35S::CiFT*α. Transgenic plants from *35S::CiTFL1* and *35S::CiBFT* showed late flowering (**Figures 6C,D**). The average time to flowering of the transgenic plants ranged from 41.1 to 52.1 days, while that of the control plants was 34.1 days. The average number of leaves at flowering ranged from 15.8 to 20.1 in the transgenic plants and was 12.1 in the control plants (**Figure 6J**). Two transgenic lines from *35S::CiTFL1* plants flowered significantly later than the control plants (80- to 120-day delay in flowering, **Figure 6E**). It is worth noting that some lines showed flowering defects including alterations in floral organ number, pale green sepals, vestigial petals, and persistent sepals around the fruit (**Figures 6F–H**). However, no difference in the appearance of flowering time, flower and inflorescences was observed between *35S::CiBFT* and control plants. Twenty-six plants were obtained in the T_1_ generation. However, *CiMFT* did not affect flowering and flower inflorescences in transgenic *Arabidopsis* (data not shown).

### Isolation, Structure Analysis, and Expression Patterns of *CiFT*, *CiBFT*, and *CiTFL1* Promoter

To further study the expression of *CiFT, CiTFL1*, and *CBFT*, the promoters (about 1.5–2.0 kb) of the three genes were amplified from lemon by using the genome walking method. We confirmed that these sequences were the promoters of the three genes by comparing them with the sweet orange genome (Xu et al., 2013). The 5′ upstream region of the above three genes were analyzed by using PLACE software. The results showed that the common elements were found in these promoters such as the putative transcriptional start site, TATA box, CAAT box, and different binding motifs (hormone response elements, light regulation, and drought response elements) (**Supplementary Table S3**). To examine the spatial patterns of *CiFT, CiTFL1*, and *CBFT*, we generated transgenic *Arabidopsis* with the *GUS* reporter gene driven by these putative promoters. Consequently, we obtained >30 independent transgenic lines for each promoter.

**FIGURE 7:**
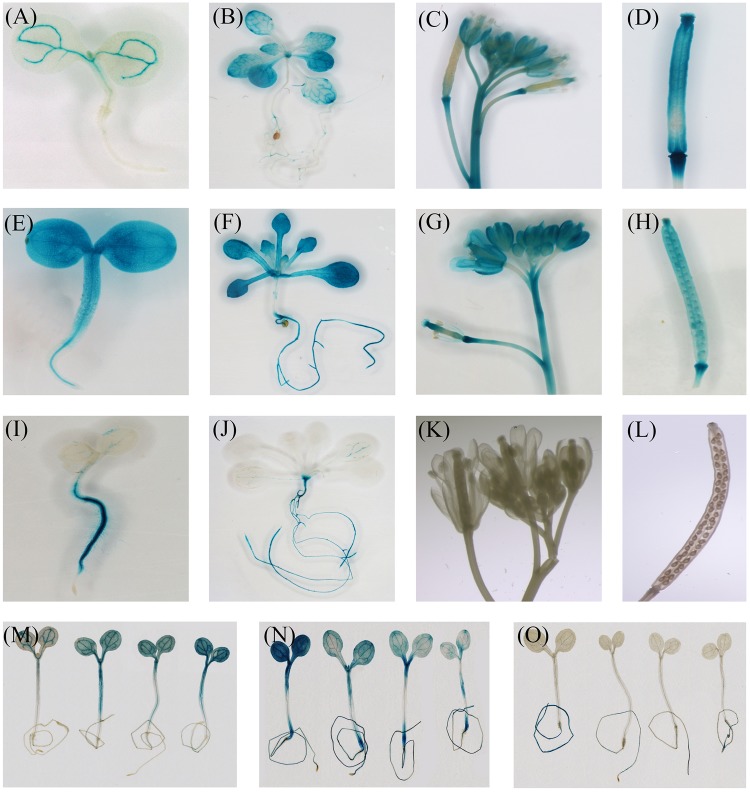
Histochemical localization of *GUS* in transgenic *Arabidopsis*. Histochemical *GUS* staining of the *CiFT*, *CiBFT*, *CiTFL1* promoters (**A,B**: *CiFT*; **E,F**: *CiBFT*; **I,J**: *CiTFL1*) at 7 and 28 days after germination (DAG). Histochemical *GUS* staining of the *CiFT*, *CiBFT*, *CiTFL1* promoters (**C**: *CiFT*; **G**: *CiBFT*; **K**: *CiTFL1*) in flower of transgenic *Arabidopsis*. Histochemical *GUS* staining of three promoters (**D**: *CiFT*; **H**: *CiBFT*; **L**: *CiTFL1*) in fruit of transgenic *Arabidopsis.* Histochemical localization of *GUS* activity in untreated and water deficit-treated transgenic *Arabidopsis* (**M**: *CiFT*; **N**: *CiBFT*; **O**: *CiTFL1*).

Histochemical staining indicated that the expression of *CiFT* promoter was seen first in the vascular tissues of cotyledons. With the development of plants, *GUS* staining was present throughout the whole plant except roots. In inflorescences, *CiFT* promoter was also detected in the vascular tissues of pedicels and floral organs but not in the inflorescence meristem (**Figures 7A–D**). The *GUS* signal from *CiTFL1* promoter can be observed at different stages. In juvenile transgenic plants, *GUS* staining was present throughout the whole plant. Further analysis of *GUS* activity in various organs of transgenic plants revealed that *GUS* staining was observed in rosette leaf, cauline leaf, flowers, silique pods and roots (**Figures 7E–H**). *GUS* expression gradually decreased at the adult stage. The *CiBFT* promoters were found only in the cell elongation zone and around the vascular bundles of the roots except root tip in the entire life cycle of transgenic plants. The same pattern is observed in lateral roots (**Figures 7I–L**). Closer analysis revealed low *GUS* activity in the leaves. In addition, the expression of *CiFT*, *CiTFL1*, and *CiBFT* promoter response to water deficit treatments was also investigated; the expression of *CiBFT* and *CiTFL1* promoters was decreased and that of *CiFT* promoter was increased under water deficit conditions (**Figures 7M–O**).

## Discussion

Molecular mechanisms underlying vernalization, photoperiod, circadian clock, and GA control of flowering time have been elucidated in annual and perennial plants, but how plants regulate flowering in response to water deficit, remains poorly understood. Because plant hormones involved in diverse biological processes of biotic and abiotic stress, so it is not surprising that associations exist between water deficit-regulated flowering and plant hormones (Koshita et al., 1999; Kazan and Lyons, 2016). In past studies, to elucidate their roles in flower-bud induction, plant hormones have been applied exogenously in crops (Dokoozlian and Peacock, 2001; Lenahan et al., 2006). However, the role of ABA is poorly understood in the regulation of plant flowering. Various studies have clearly shown that ABA plays an key role in stress responses of higher plants, plants accumulate high levels of ABA accompanied under water deficit condition (Duque and Setter, 2013; Shanker et al., 2014). ABA promotes transcriptional up-regulation of *FT*, *TSF*, and *SOC1*, leading to flowering only under long days (Riboni et al., 2013). Recent data also indicated that ABA involved in the photoperiodic induction of flowering in *Pharbitis nil* seedlings (Wilmowicz et al., 2008). In this study, ABA content significantly increased in buds during the water deficit process. The buds were in an undetermined state and floral primordia were not observed before the beginning of water deficit. When the treatment began, differentiation occurred rapidly and produced the primordia of sepal. Furthermore, although no significant differences were observed between ABA-treated trees and the control, the expression of *CiFT* was induced by ABA treatment. Therefore, although there was no direct evidence connecting the ABA contents and floral induction in lemon, endogenous ABA might be one of the key factors during floral inductive water deficits, because different levels were observed during the flower bud induction period.

Gibberellins has been extensively reported in inhibiting floral initiation of woody plants (Lee and Safe, 2008). Berthelsen et al. (2002) suggested that GAs act indirectly on the floral process by delaying bud formation. In this study, the number of flowers was increased with PBZ treatment compared to the control. These results indicated that water deficit may inhibit endogenous GA production. Interestingly, the IAA content was induced immediately at the beginning of water deficit and most maintained high expression levels after releasing from water deficit. Therefore, the role of endogenous IAA may be maintaining the necessary vegetative growth during floral inductive water deficits. In addition, according to the overview of the transcriptome profiles, 1638 genes were differentially expressed during the water deficit process. The KEGG analysis shows that the genes responding to water deficit are mainly related to plant hormone signal transduction, biosynthesis of secondary metabolites, as well as starch and sucrose, which has been suggested to be associated with floral initiation and flower development. Traditionally, sugars were regarded as energy supply, but recently sugars have been suggested to serve as signals during plant development (Lebon et al., 2008). Model plants containing mutations in starch biosynthesis or sugar transporter genes exhibit regulated flowering (Corbesier et al., 1998; O’Hara et al., 2013). These data indicated that plant hormones, starch, and sucrose may be involved in flowering induction under water deficit conditions.

Alternative splicing is involved in some important development processes in plants, such as flowering (Eckardt, 2002). To explore potential AS events during the water deficit process, we preformed computational analyses to determine AS junctions. Overall, our data indicated that 27% of lemon genes undergo AS, similar to the rate previously reported for trifoliate orange (Ai et al., 2012). However, this number is significantly lower than predicted in *Arabidopsis*, in which AS is estimated to occur in 61% of all genes (Wang and Brendel, 2006; Marquez et al., 2012). As previously reported in *Arabidopsis* and rice (Wang and Brendel, 2006), intron retention is the primary type of AS. This is different from trifoliate orange AS events in which the alternative 3′ spliced site is the most prevalent mechanism (Ai et al., 2012). The differences in AS frequency and alternative splice type between lemon and trifoliate orange may reflect underlying differences in pre-mRNA splicing regulation under stress conditions. It is also very possible that part of the differences has to do with the methods software settings and defaults used to construct the transcript build and AS analysis. In addition, trifoliate orange is phylogenetically fairly distant from lemon, and from different climate zones, that maybe another contributor to the differences. Many flowering-related genes of AS events were involved in floral inductive. For example, a previous study indicated that AS of trifoliate orange *FCA* has functional significance related to its role in the floral transition (Ai et al., 2016). AS of *FLC* was also associated with the transition from juvenile to mature trees in trifoliate orange (Zhang et al., 2009a). These AS events of flowering genes were also involved in water deficit-regulated flowering. The lemon *FT* ortholog has AS events, consistent with previous reports on *FT* of London plane (Zhang J. et al., 2011). Furthermore, the expression patterns of the different *CiFT* AS transcripts (*CiFT*α and *CiFT*β) were related to floral induction under water deficit treatment and hormone treatment conditions. Plants over-expressing *CiFT*α and *CiFT*β flowered earlier than the control in transgenic *Arabidopsis*. These results suggest that *CiFT* acts as a floral inducer during floral inductive water deficit.

In model plants, *GI* is a key regulator of the drought-escape response and promotes flowering via the photoperiod and circadian pathways (Mishra and Panigrahi, 2015). Under long-days condition, water deficit stress triggers transcriptional induction of *FT* and *TSF* in a manner dependent on GI and ABA. Under short days condition, water deficit and ABA are thought to activate floral suppressors, inhibiting the expression of *FT* and *TSF* (Riboni et al., 2013). In this study, the expression level of *GI* and the ABA content significantly increased during the water deficit treatment. Therefore, the regulatory mechanism of water deficit-regulated flowering in lemon may be similar to that of model plants. However, lemon *FT* and *TSF* were not found among the DEGs; only two other PEBP family members (*MFT* and *BFT*) were differentially expressed. This might be explained by the fact that FT protein is a mobile signal synthesized in leaves and transported to the shoot apical meristem (Turck et al., 2007; Zhang et al., 2009b). In order to confirm this speculation, the expression levels of *CiFT* were investigated in leaves. The results indicated that the levels of *CiFT* and promoter increased under water deficit conditions. This increased *CiFT* was correlated with more flowers being formed in trees under water deficit condition than control trees. Therefore, the accumulation of *CiFT* seems to be sensitive to water deficit. To this effect, buds from GA_3_-treated trees showed significantly decreased *CiFT* compared to the control trees.

In contrast, we were not able to identify *TSF* homologs gene in the citrus genome by using several *TSF*-like sequences from other plants as a query. In model plants, Yamaguchi et al. (2005) indicated that *TSF* gene acts as a floral pathway integrator and promotes flowering redundantly with *FT*. These results indicated that the transcripts *CiFT*β may play a similar role during the water deficit process. Our previous work revealed that the promotion of trifoliate orange flowering by *FT* and *TFL1* was largely achieved through up-regulation of *FT* and down-regulation of *TFL1* in trifoliate orange (Zhang et al., 2009b). However, we were unable to detect the expression of *TFL1* because of its low expression. In our previous study, the expression of *TFL1* also was not detected in the vegetative and flower buds of trifoliate orange by RNA sequencing (Zhang J.Z. et al., 2011). Therefore, the expression of *CiTFL1* was investigated during floral inductive water deficits. In treated bud samples, we noted reduced expression of *CiBFT* and *CiTFL1* in trees under water deficit condition but increased accumulation both control and GA-treated trees, indicating that water deficit acts as a negative regulator of the two genes. The promoters of *CiFT*, *CiBFT*, and *CiTFL1* were regulated by water deficit treatment. The promoters of *CiBFT* and *CiTFL1* were down-regulated and the promoter of *CiFT* was up-regulated under water deficit conditions. These results further indicated that the three genes play an important role during water deficit.

In addition, several putative homologs of *Arabidopsis* flowering-related and floral identity genes were found among DEGs under water deficit conditions besides members of the PEBP family and *GI*, such as *SPL6/8/9/13*, *GI*, *ELF3*, and *ELF4*. Some of these genes are required for light signal transduction pathways, while others encode components of the day length response or are involved in circadian clock function (Khan et al., 2014). For example, *ELF3* has been implicated as a suppressor of light signaling to the circadian clock (Dixon et al., 2011). In model plants, *ELF4* as a signaling intermediate in promotion of circadian clock function and photoperiod perception (Khanna et al., 2003). *GI* and *ELF4* exhibit differential phase-specific genetic influences over a diurnal cycle in *Arabidopsis* (Kim et al., 2012). *SPL* proteins constitute a diverse family of TFs that play fundamental roles in plant growth and development such as flowering (Preston and Hileman, 2013). In this study, the expression patterns of *CiELF3*, *CiELF4*, *CiGI*, and *CiSPLs* were closely correlated with floral induction during the water deficit process. However, no differences in gene expression were found throughout the experiment by GA3, NAA, ABA, and PBZ treatments. Although we could not identify the exact role of these genes, our expression data indicated that water deficit is essential for the up-regulation of these genes, suggesting that they may play an important role during floral inductive water deficits.

## Conclusion

**FIGURE 8:**
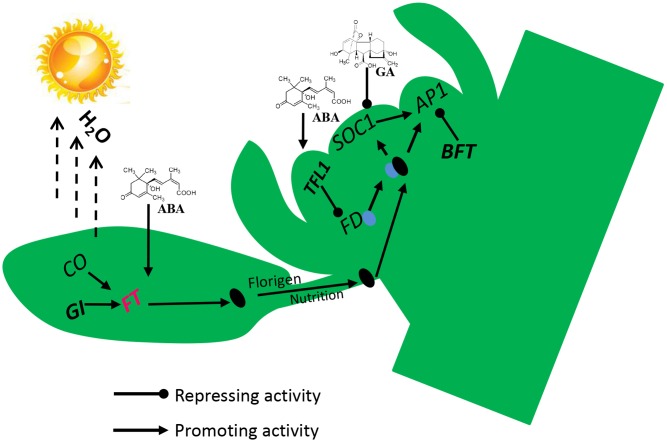
Proposed flowering model for molecular events occurring in lemon buds during floral inductive water deficits. Arrow-ended and blunt-ended lines represent process induction and suppression, respectively.

To identify the physiological and molecular mechanism of lemon flowering during floral inductive water deficits, we analyzed the morphology, cytology, and gene expression profiles of buds and performed functional analysis of some of the key flowering genes. Our results reveal an interaction between water deficit and hormones in the activation of the florigen related genes, with the process requiring GI and the hormones ABA, GAs, and IAA (**Figure 8**). Before water deficits, ABA, GAs, and IAA are produced in the buds, and some stimulus coming from the lemon leaves allows the expression of the floral bud potential, with the high level of GAs and IAA interfering with it. Therefore, the buds do not form floral buds and continue to produce vegetative buds. At the beginning of water deficit treatment, GAs and IAA are decreased and ABA is rapidly increased in the buds, GI protein directly binds to the *FT* promoter, and *FT* is up-regulated, and then florigen and nutrients are gradually transported from leaves to the bud. FT protein is transported to the bud, where FT protein interacts with FD protein (a bZIP TF required for the transition to flowering promoted by FT) by competing against TFL1 protein. *TFL1* was reduced in buds by water deficit, and FD converts FT into a strong activator, which binds to the *AP1* promoter of floral meristem identity genes. *BFT* was also down-regulated by water deficit and reduced *BFT* levels further trigger *AP1* activation, which in turn contributes to up-regulation of floral organ gene. Finally, nutrients begin to accumulate in the lateral bud for flower bud differentiation and flowering.

## Data Archiving Statement

The RNA-Seq data has been submitted to Gene Expression Omnibus (GEO) under accession number GSE90596. *CiMFT* (MF069597), *CiBFT* (MF069598), *CiTFL1* (MF069599), *CiFT*α (MF069600), and *CiFT*β (MF069601) have been deposited with the GenBank database. Meanwhile, the *CiMFT* (MF069602), *CiBFT* (MF069603), *CiTFL1* (MF069604), *CiFT* (MF069605) promoters have been also deposited with the GenBank database.

## Author Contributions

J-XL, J-ZZ, JZ, J-JZ, J-QY, C-XH, and C-GH conceived the research plan and supervised the experiments; H-BH, X-JH, J-YG, Y-XD, and JZ performed the experiments and analyzed the data; J-XL, J-JZ, and JZ-Z drafted the manuscript. All authors read and approved the final manuscript.

## Conflict of Interest Statement

The authors declare that the research was conducted in the absence of any commercial or financial relationships that could be construed as a potential conflict of interest.

## Footnotes

1 http://citrus.hzau.edu.cn/orange/

## Supplementary Material

**FIGURE S1** | Gene ontology analysis of differentially expressed genes.

**FIGURE S2** | Pathway enrichment analysis of differentially expressed genes using the program KEGG pathway.

**FIGURE S3** | Phenotypic characteristics of PBZ-treated and the control plants. **(A)** The PBZ-treated and control plants after resumption of irrigation; **(B)** Effect of PBZ on flowering of lemon. The data were processed using one-way analysis of variance (ANOVA), and statistical differences were compared based on Student’s *t*-test, taking *P* < 0.01 as ^∗∗^.

**FIGURE S4** | Evidence for alternative splicing of *CiFT*β in transgenic *Arabidopsis.* The multiple sequence alignment was performed with Clustal X.

**TABLE S1** | Primer sequence information.

**TABLE S2** | Analysis of the 1638 differentially expressed genes.

**TABLE S3** | *Cis*-elements in the *CiFT*, *CiBFT*, and *CiTFL1* promoters predicted by PlantCARE database analysis. Positions are relative to start codon. The orientation of the motifs is indicated (+, forward; -, reverse).
