# Supplementary figures and images for: Identification of Genes Associated with Lemon Floral Transition and Flower Development during Floral Inductive Water Deficits: A Hypothetical Model
Source: Front Plant Sci. 2017 Jun 13;8:1013. doi: 10.3389/fpls.2017.01013 (PMC5468436; doi:10.3389/fpls.2017.01013)

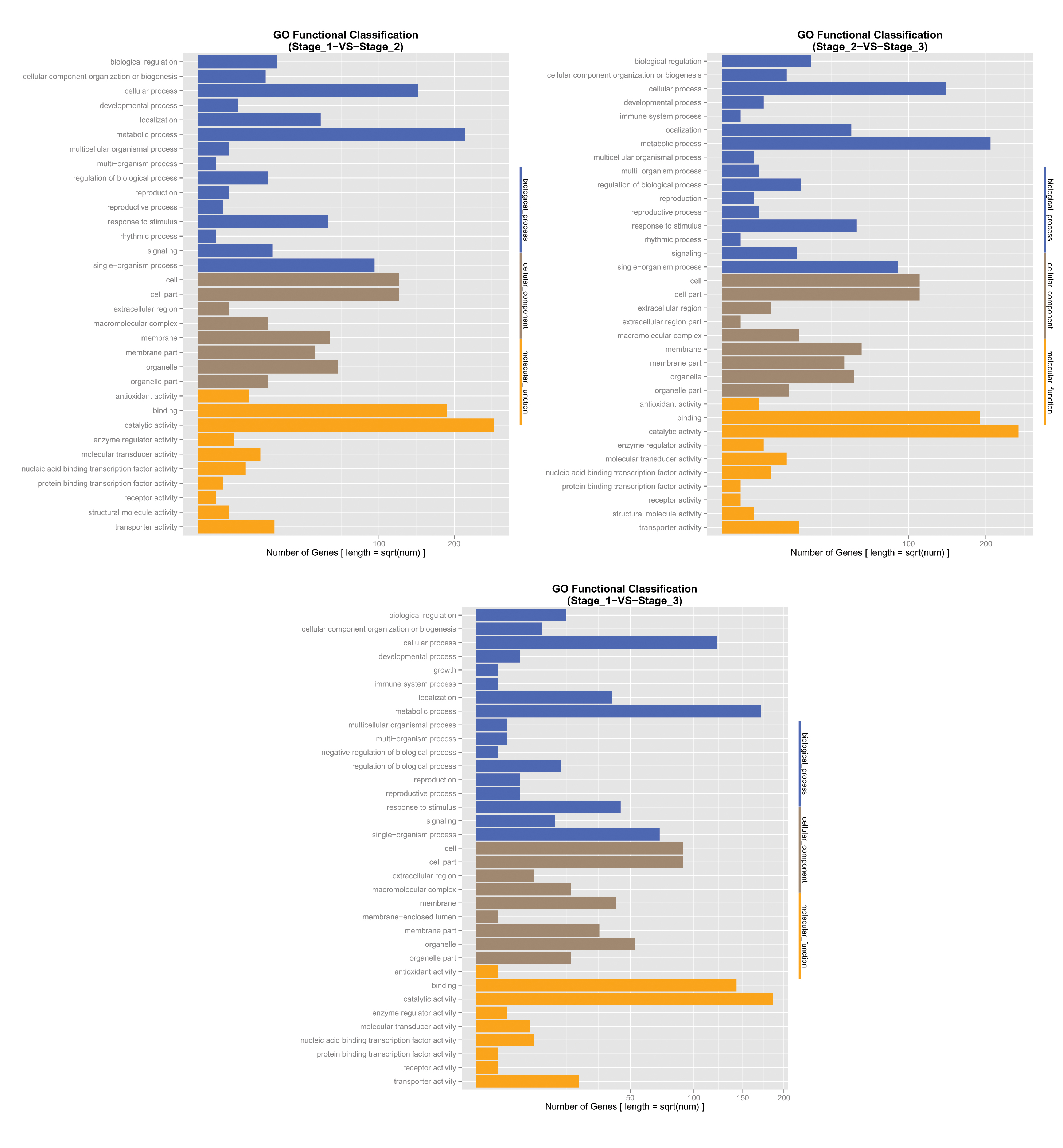

Supplement: Supplementary file 1 [file Image_1.JPEG]

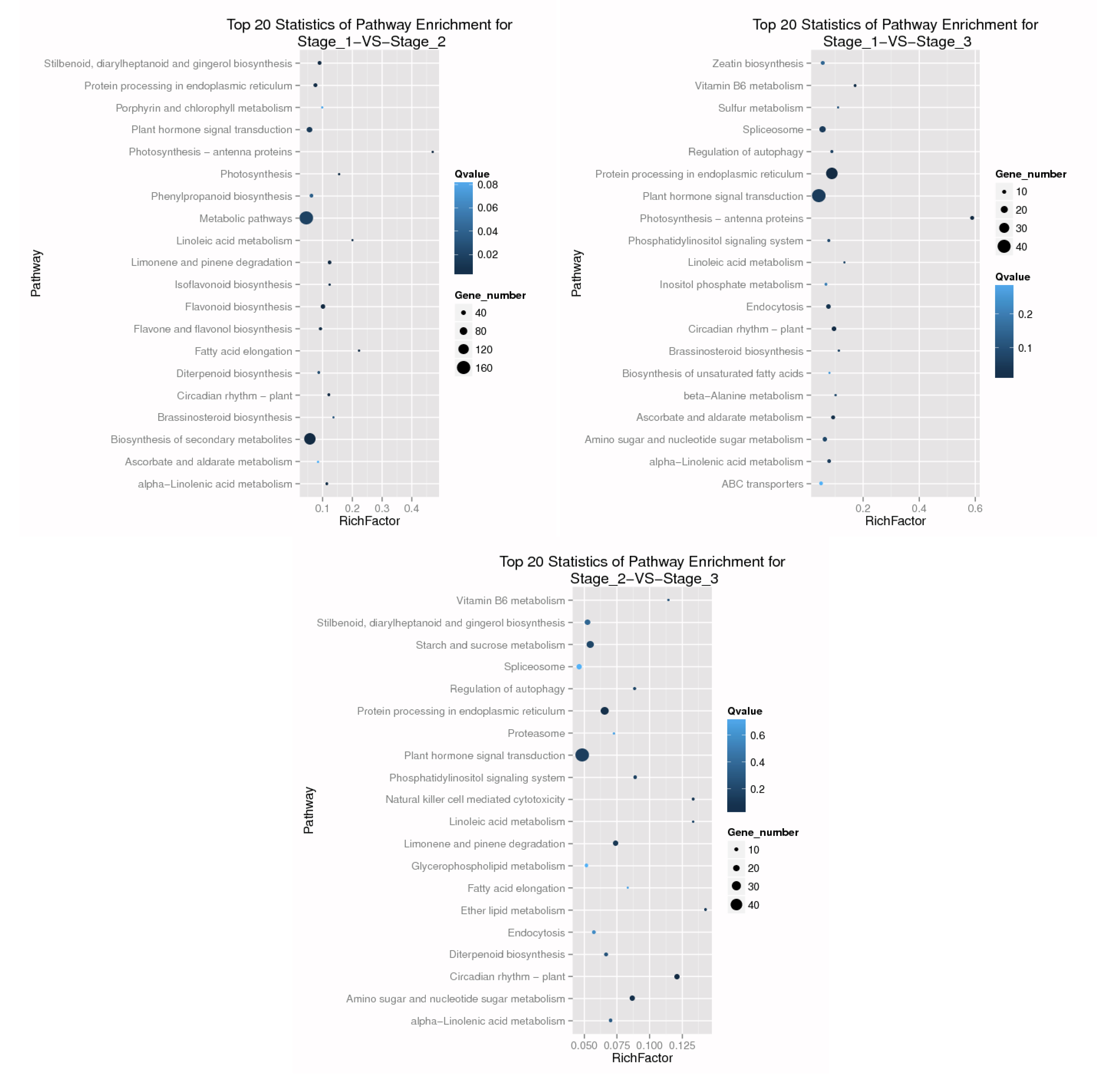

Supplement: Supplementary file 2 [file Image_2.JPEG]

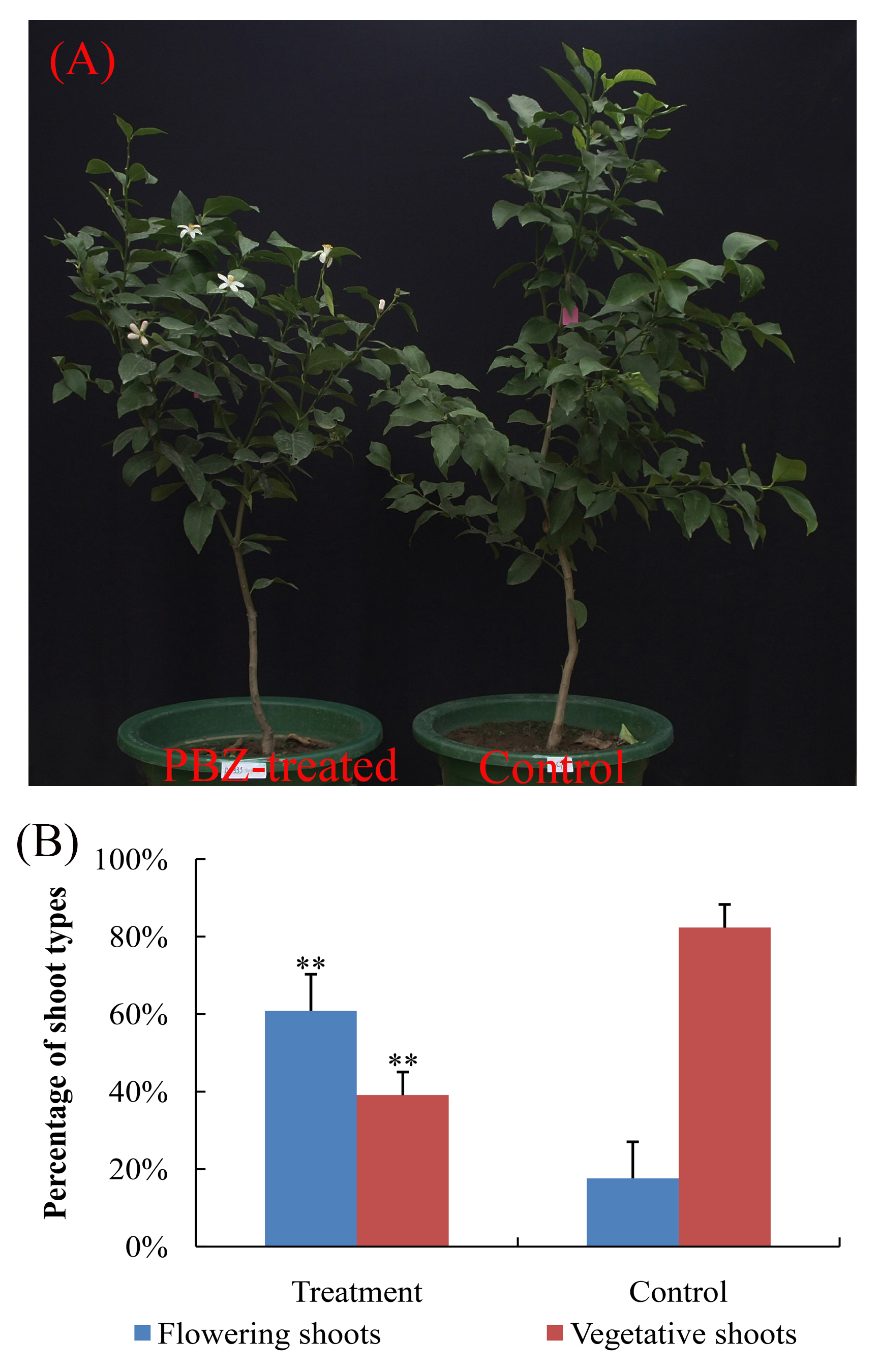

Supplement: Supplementary file 3 [file Image_3.JPEG]

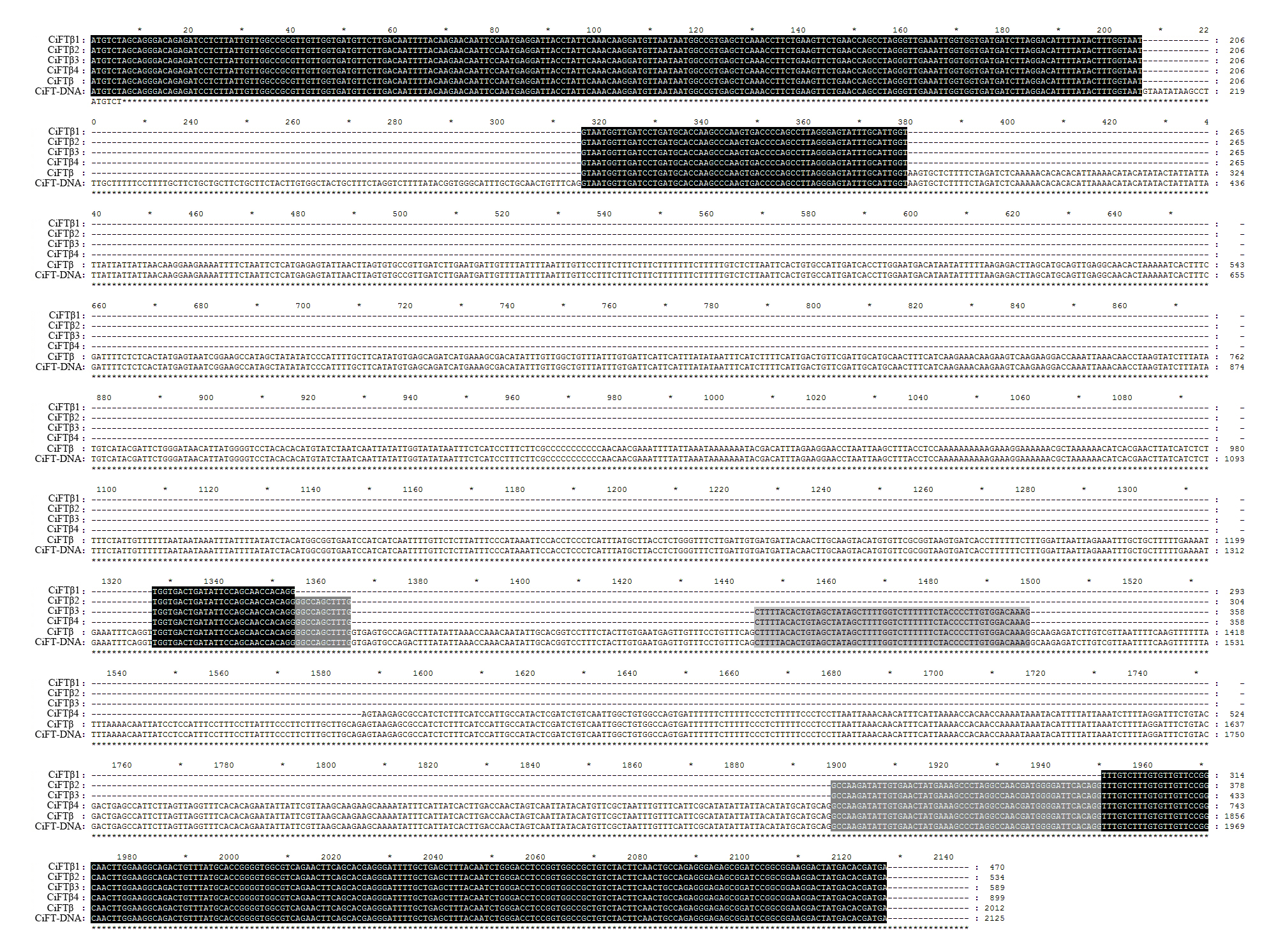

Supplement: Supplementary file 4 [file Image_4.JPEG]
